# The validity and consistency of continuous joystick response in perceptual decision-making

**DOI:** 10.3758/s13428-019-01269-3

**Published:** 2019-07-03

**Authors:** Maciej J. Szul, Aline Bompas, Petroc Sumner, Jiaxiang Zhang

**Affiliations:** Cardiff University Brain Research Imaging Centre, School of Psychology, Cardiff University, Cardiff, UK

**Keywords:** Joystick trajectory, Decision-making, Computational modeling, Behavioral experiments, Drift-diffusion model

## Abstract

A computer joystick is an efficient and cost-effective response device for recording continuous movements in psychological experiments. Movement trajectories and other measures from continuous responses have expanded the insights gained from discrete responses (e.g., button presses) by providing unique information about how cognitive processes unfold over time. However, few studies have evaluated the validity of joystick responses with reference to conventional key presses, and how response modality can affect cognitive processes. Here we systematically compared human participants’ behavioral performance of perceptual decision-making when they responded with either joystick movements or key presses in a four-alternative motion discrimination task. We found evidence that the response modality did not affect raw behavioral measures, including decision accuracy and mean response time, at the group level. Furthermore, to compare the underlying decision processes between the two response modalities, we fitted a drift-diffusion model of decision-making to individual participants’ behavioral data. Bayesian analyses of the model parameters showed no evidence that switching from key presses to continuous joystick movements modulated the decision-making process. These results supported continuous joystick actions as a valid apparatus for continuous movements, although we highlight the need for caution when conducting experiments with continuous movement responses.

**Electronic supplementary material:**

The online version of this article (10.3758/s13428-019-01269-3) contains supplementary material, which is available to authorized users.

Discrete key presses on a keyboard or button box have been the long-standing response modality in computer-based experiments in psychology, from which on/off responses and response time (RT) are commonly measured. Developments in computer and electronic technology have improved the accessibility of other devices that are capable of recording continuous responses—for example, a joystick, computer mouse, motion sensor, or robotic arm (Koop & Johnson, 2011; O’Hora, Dale, Piiroinen, & Connolly, 2013). In addition to the standard behavioral measures available from key presses, continuous responses enable further inferences from movement trajectories. However, to utilize the full capacity of continuous response recording, we need to ensure that the experimental results from these devices are consistent with, or generalizable to, the findings from conventional response modalities such as key presses. In the present study, we addressed this issue by comparing behavioral performance between joystick movements and key presses in a perceptual decision-making task. Using computational modeling of behavioral data, we further compared the decision-making processes from the two response modalities.

## Continuous and discrete responses in experimental psychology

Continuous responses can offer theoretical and practical advantages in experiments. First, although a discrete response is consistent with the assumption of sequential stages of cognition and motor outputs, a growing number of studies have suggested a continuous and parallel flow of information between the brain systems involved in sensory, cognitive, and motor processes (Cisek & Kalaska, 2005; Spivey, Grosjean, & Knoblich, 2005). Continuous responses can capture the dynamics of these multiple mental processes, as well as the transitions between them (Resulaj, Kiani, Wolpert, & Shadlen, 2009). Second, in experiments involving clinical populations, it can be difficult for patients to make discrete responses accurately on a keyboard, especially in patients with dementia or parkinsonism. Patients with motor function impairments (e.g., tremor, apraxia, or loss of dexterity) often omit button presses, press the button too early or too late, press wrong buttons accidentally, or are confused by the response-button mapping. This limitation may result in a significant amount of experiment data being rejected in some studies (Wessel, Verleger, Nazarenus, Vieregge, & Kömpf, 1994), whereas continuous responses with natural movements can be well-tolerated in patients (Limousin et al., 1997; Strafella, Dagher, & Sadikot, 2003)

The trajectories of continuous movements contain rich spatiotemporal information about the action and provide unique insights into how cognitive processes unfold in time (Freeman, Dale, & Farmer, 2011; Song & Nakayama, 2009). In continuous reaching, movement trajectories showed that human participants can initiate a reaching action prior to when the target becomes fully available and can select from competing action plans at a later stage (e.g., Chapman et al., 2010; Gallivan & Chapman, 2014). In perceptual decision-making, movement trajectories from joysticks and other similar devices have been successfully used to investigate the cognitive processes underlying changes of mind (Resulaj et al., 2009), error correction (Acerbi, Vijayakumar, & Wolpert, 2017), and subjective confidence (van den Berg et al., 2016) that would otherwise be difficult to study with key presses.

### A comparison between response modalities

To extend the currently available experimental findings to other devices, it is necessary to assess the consistency of performance between response modalities. More importantly, characterizing the consistency between response modalities may help us understand the interdependence of cognitive processes and motor systems. For example, in decision-making tasks, comparisons between saccadic eye movements and manual responses have suggested that a domain-general decision mechanism operates, regardless of response modality (Gomez, Ratcliff, & Childers, 2015; Ho, Brown, & Serences, 2009), and that the apparent difference in response speed is accounted for by the neuroanatomical distinctions in saccadic and manual networks (Bompas, Hedge, & Sumner, 2017).

In the present study we aimed to examine the validity and consistency of continuous joystick responses versus discrete button presses in perceptual decision-making. Participants performed a four-alternative motion discrimination task (Churchland, Kiani, & Shadlen, 2008) with two levels of perceptual difficulty. The task was to indicate the coherent motion direction from a random-dot kinematogram, a standard psychophysical stimulus for visual perceptual decision (Fredericksen, Verstraten, & Van De Grind, 1994; Lappin & Bell, 1976; Pilly & Seitz, 2009; Ramachandran & Anstis, 1983; Watamaniuk, Sekuler, & Williams, 1989). In two counterbalanced sessions, the participants indicated their decisions with either joystick movements or key presses. The joystick response was to move the lever from its neutral position toward one of the four cardinal directions, aligned to the coherent motion direction, and the corresponding key press was one of the four arrow keys on the keyboard. We compared the raw behavioral performance (decision accuracy and mean RTs) between the two response modalities and between the two levels of task difficulty. From the continuous movement trajectories, we also examined whether the joystick-specific measures were consistent between movement directions (i.e., trajectory length, peak velocity, and acceleration time).

To assess whether the response modality affected the decision-making process, we fitted a drift-diffusion model (DDM; Gold & Shadlen, 2007; Ratcliff, Smith, Brown, & McKoon, 2016) to the individual participants’ behavioral data and compared the model parameters derived from the joystick and keyboard sessions. The DDM belongs to a family of sequential-sampling models of RT. These models assume that the decision process is governed by the accumulation of noisy sensory evidence over time until a threshold is reached (Bogacz, Brown, Moehlis, Holmes, & Cohen, 2006; Ratcliff & Smith, 2004), consistent with electrophysiological (Britten, Shadlen, Newsome, & Movshon, 1992; Churchland et al., 2008; Hanks, Kiani, & Shadlen, 2014; Huk & Shadlen, 2005; Shadlen & Newsome, 2001) and neuroimaging (Heekeren, Marrett, & Ungerleider, 2008; Ho et al., 2009; Zhang, Hughes, & Rowe, 2012) evidence on the identification of neural accumulators in the frontoparietal cortex. In the present study we used the DDM to decompose the observed RT distributions and accuracy into three main model components: decision threshold for the amount of evidence needed prior to a decision, drift rate for the speed of evidence accumulation, and nondecision time to account for the latencies of stimulus encoding and action initiation (Karahan, Costigan, Graham, Lawrence, & Zhang, 2019; Ratcliff & McKoon, 2008; Wagenmakers, 2009; Zhang, 2012). The latter parameter is of interest, because one might expect to find a difference in the latency distributions of action initiation between joystick movements and key presses.

Our findings demonstrated that when human participants used ballistic movements to respond with a joystick, their behavioral performance was modulated by task difficulty and was similar to that from key presses during the same perceptual task. Further computational modeling analysis showed no evidence of a change in any model parameter when switching between response modalities. As such, we concluded that joystick movement is a valid response modality for extending discrete actions to continuous behavior in psychological experiments, although participants might exhibit differences in movement trajectory measures for different directions.

## Method

### Participants

Twenty-one participants (14 females, seven males; age range 18–24 years, *M* = 20.43 years, *SD* = 2.91 years) took part in the study following written informed consent. All but three were right-handed. All the participants had normal or corrected-to-normal vision, and none reported a history of motor impairments or neurological disorders. The study was approved by the Cardiff University School of Psychology Ethics Committee.

### Apparatus

The experiment was conducted in a behavioral testing room with a dimmed light. The stimuli were displayed on a 22-in. CRT monitor with 1,600 × 1,200 pixel resolution and 85-Hz refresh rate. A chin rest was used to maintain the viewing distance and position. A joystick (Extreme 3D Pro Precision, Logitech International S.A., Switzerland) was used to record movement trajectories at 85 Hz in the joystick session. The experimental setup for the joystick and keyboard sessions is illustrated in Supplementary Fig. 1. The joystick handle could move nearly freely, with little resistance from its neutral position within the 20% movement radius. Beyond the 20% radius, the resistance during joystick movement was approximately constant. A standard PC keyboard was used to record key presses. The experiment was written using PsychoPy 1.85.4 library (Peirce, 2009).

### Stimuli

In both the joystick and keyboard sessions, a random-dot kinematogram was displayed within a central invisible circular aperture of 14.22° diameter (visual angle). White dots were presented on a black background (100% contrast), with a dot density of 27.77 dots per degree^2^ per second and a dot size of 0.14°. As in previous studies (Britten et al., 1992; Pilly & Seitz, 2009; Roitman & Shadlen, 2002; Shadlen & Newsome, 2001; Zhang & Rowe, 2014), we introduced coherent motion information by interleaving three uncorrelated sequences of dot positions across frames at 85 Hz. In each frame, a fixed proportion (i.e., the motion coherence) of dots were replotted at an appropriate spatial displacement in the direction of the coherent motion (51.195°/s velocity), relative to their positions three frames earlier, and the rest of the dots were presented at random locations within the aperture. The signal dots had a maximum lifetime of three frames, after which they were reassigned to random positions. The coherent motion direction in each trial was set in one of the four cardinal directions (0°, 90°, 180°, or 270°).

### Task and procedure

Each participant took part in two experimental sessions using keyboard or joystick as a response modality. The order of response modality was counterbalanced across participants. In both sessions, participants performed a four-alternative motion discrimination task, indicating the coherent motion direction from four possible choices (0°, 90°, 180°, or 270°). Each session comprised 960 trials, which were divided into eight blocks of 120 trials. Each block had 15 repetitions of each of the four motion directions and two difficulty conditions. The motion coherence was set to 10% in the “Difficult” condition and 20% in the “Easy” condition. Feedback on the mean decision accuracy was provided after each block. The order of the conditions was pseudo-randomized across sessions and participants, ensuring that the same direction and difficulty condition did not occur in four consecutive trials. In the keyboard session, the participants responded with four arrow keys corresponding to the coherent motion directions (right, 0°; up, 90°; left, 180°; and down, 270°). In the joystick session, the participants were instructed to indicate the motion direction with an appropriate joystick movement from the joystick’s central position toward one of the four edges (right, 0°; up, 90°; left, 180°; and down, 270°).

**Fig. 1:**
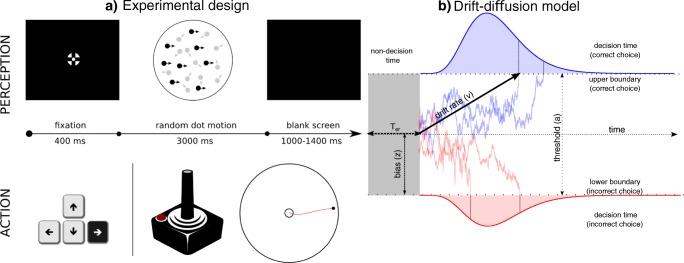
Behavioral paradigm and the drift-diffusion model (DDM). **a** The structure of a single trial of the experiment. A fixation screen was presented for 400 ms, after which the random-dot kinematogram was presented for a maximum of 3,000 ms or until response. The intertrial interval was randomized between 1,000 and 1,400 ms. Participants were instructed to indicate the direction of the coherent motion direction (0°, 90°, 180°, or 270°) using the joystick or keyboard, in two counterbalanced sessions. **b** The DDM and examples of evidence accumulation trajectories. The parameter (*a*) indicates the distance between the correct and incorrect decision thresholds. The drift rate (*v*) represents the speed of evidence accumulation, and its magnitude is determined by the quality of the evidence. A positive *v* indicates that, on average, the accumulation of sensory evidence is toward the correct decision threshold. The starting point (*z*) represents the response bias toward one of the two thresholds. The nondecision time (*T*_er_) represents the latencies of nondecision processes, illustrated by the gray area beside the decision time distribution in the figure. The diffusion process starts at the starting point (*z*) until the accumulated evidence reaches one of the two thresholds. If the accumulated evidence reaches the correct (upper) threshold (blue trajectories), the model predicts a correct response. Because of noise, the accumulated evidence might reach the incorrect (lower) threshold (red trajectories), so the model would predict an incorrect response. The predicted single-trial response time is the sum of the duration of the evidence accumulation (decision time) and the nondecision time *T*_er_

Every trial started with a 400-ms fixation period (Fig. 1a). The random-dot kinematogram appeared after the fixation period for a maximum of 3,000 ms or until response. In the keyboard session, the stimuli disappeared after a button press. In the joystick condition, the stimuli disappeared when the participants stopped the joystick movement. The chosen stopping rule was when the joystick position did not change in the last four sampling points and its position was outside of the 20% motion radius. After response, a blank screen was presented as the intertrial interval, with a duration uniformly randomized between 1,000 and 1,400 ms.

The RT in the keyboard session was defined as the latency between the onset of random-dot kinematogram and the time of key press. In the joystick session, the RT was defined as the duration between the onset of the random-dot kinematogram and the first time when the joystick’s position left the 20% movement radius from its neutral position. It coincided with the first noticeable increase in the velocity of the movement from the stimulus onset. Participants’ choice in the joystick session was one of the four cardinal directions (i.e., 0°, 90°, 180°, or 270°) closest to the last position of the joystick.

### DDM analysis

We fitted the DDM to each participant’s response time distributions and accuracy. The DDM decomposes the behavioral data into four key model parameters (Ratcliff & McKoon, 2008). The *decision threshold* (*a*) denotes the distance between the two decision boundaries. The *mean drift rate* (*v*) denotes the strength of sensory information. The *starting point* (*z*) denotes the response bias toward one of the two alternatives. The *nondecision time* (*T*_er_) denotes the latencies of stimulus encoding and response initiation. In addition, the DDM can be extended to include trial-by-trial variability in drift rate *s*_*v*_ and nondecision time *s*_*t*_, which improves the model fit to the data (Ratcliff & McKoon, 2008). The DDM predicts the decision time as the duration of the accumulation process and the observed RT as the sum of the decision time and *T*_er_(Fig. 1b).

As in previous studies (Churchland et al., 2008), we simplified the four-alternative forced choice task in the present study to a binary decision problem for model fitting. This was achieved by separately grouping trials with correct responses and trials with incorrect responses. The behavioral task was then reduced to a binary choice between a correct and an incorrect alternative. We used the hierarchical drift-diffusion model (HDDM) toolbox to fit the behavioral data (Wiecki, Sofer, & Frank, 2013). The HDDM implemented a hierarchical Bayesian model (Vandekerckhove, Tuerlinckx, & Lee, 2011) for estimating the DDM parameters, which assumes that the model parameters for individual participants are sampled from group-level distributions at a higher hierarchy. Given the observed experimental data, the HDDM used Markov chain Monte Carlo (MCMC) approaches to estimate the joint posterior distribution of all individual- and group-level parameters. The posterior parameter distributions can be used directly for Bayesian inference (Gelman et al., 2014), and this Bayesian approach has been shown to be robust in recovering model parameters when limited data are available (Ratcliff & Childers, 2015; Wiecki et al., 2013; Zhang et al., 2016).

We applied a few constraints to the model parameters based on our task design. First, we allowed all the model parameters (*a*, *v*, *T*_er_, *s*_*v*_, and *s*_*t*_) to vary between the two response modalities. Second, the mean drift rate *v* was further allowed to vary between task difficulties (easy, difficult) and correct directions (up, down, left, and right). Third, the starting point *z* was fixed at .5, suggesting that there was no bias toward the two decision boundaries and that equal amounts of evidence were required for correct and incorrect decisions. This was because the participants did not have a priori knowledge about the correct alternative at the beginning of each trial.

We generated 15,000 samples from the joint posterior distribution of all model parameters by using MCMC sampling (Gamerman & Lopes, 2006). The initial 7,000 samples were discarded as burn-in for stable posterior estimates. Geweke diagnostic (Cowles & Carlin, 1996) and autocorrelation were used to assess the convergence of the Markov chains in the last 8,000 samples. All parameter estimates were converged after 15,000 samples.

### Data analysis

First, we used both Bayesian and frequentist repeated measures analysis of variance (ANOVA) to make inferences on behavioral measures (JASP Team, 2018). For frequentist ANOVAs, Greenhouse–Geisser correction was applied when the assumption of sphericity was violated. For Bayesian ANOVAs, we followed the standard heuristic to characterize the strength of evidence based on the Bayes factor (BF_10_; Wagenmakers, Lee, Lodewyckx, & Iverson, 2008), which can provide evidence supporting either the alternative (BF_10_ > 1) or the null (BF_10_ < 1) hypothesis. A BF_10_ between [1, 3] (or [0, 1/3]) suggests weak evidence for the alternative (or null) hypothesis. A BF_10_ between [3, 10] (or [1/10, 1/3]) suggests moderate or compelling evidence for the alternative (or null) hypothesis. A BF_10_ larger than 10 (or smaller than 1/10) suggests strong evidence for the alternative (or null) hypothesis.

Second, to quantify the difference in RT distributions between response modalities, we used the Kolmogorov–Smirnov (K–S) test (Pratt & Gibbons, 1981), a nonparametric statistical measure of difference between two one-dimensional empirical distributions.

Third, to compare a fitted DDM parameter between two conditions (e.g., between response modalities or between task difficulties), we used Bayesian hypothesis testing (Bayarri & Berger, 2004; Gelman et al., 2014; Kruschke, 2015; Lindley, 1965) to make inferences from the posterior parameter distributions, under the null hypothesis that the parameter values were equal between the two conditions.

More specifically, we first calculated the distribution of the parameter difference from the two MCMC chains of the two conditions, and we obtained the 95% highest density interval (HDI) of that difference distribution between the two conditions. We then set a region of practical equivalence (ROPE) around the null value (i.e., 0 for the null hypothesis), which encloses the values of the posterior difference that are deemed to be negligible from the null value 0 (Kruschke, 2013). In each Bayesian inference, the ROPE was set empirically from the two MCMC chains of the two conditions under comparison. For each of the two conditions, we calculated the 95% HDI of the difference distribution between odd and even samples from that condition’s MCMC chain. This 95% HDI from a single MCMC chain can be considered as negligible values around the null, because posterior samples from different portions of the same chain are representative values of the same parameter. That is, we accepted that the null hypothesis is true when comparing the difference between odd and even samples from the same MCMC chain. The ROPE was then set to the widest boundaries of the two 95% HDIs of the two conditions.

From the 95% HDI of the difference distribution and the ROPE, a Bayesian *P* value was calculated. To avoid confusion, we use *P* to refer to classical frequentist *P* values, and *P*_*P*|D_ to refer to the Bayesian *P* values based on posterior parameter distributions. If ROPE is completely contained within the 95% HDI, *P*_*P*|D_ = 1, and we accept the null hypothesis (i.e., the parameter values are equal between the two conditions). If ROPE is completely outside the 95% HDI, *P*_*P*|D_ = 0 and we reject the null hypothesis (i.e., the parameter values differ between the two conditions). If ROPE and the 95% HDI partially overlap, *P*_*P*|D_ equals the proportion of the 95% HDI that falls within the ROPE, which indicates the probability that the parameter values are *practically* equivalent between the two conditions (Kruschke & Liddell, 2018).

## Results

### Behavioral results

**Fig. 2:**
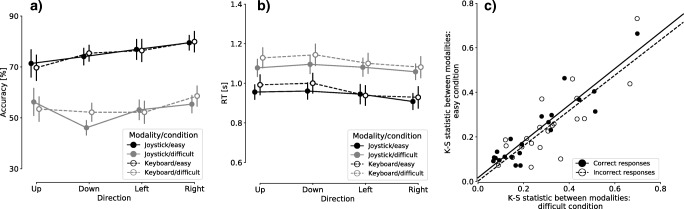
Behavioral results in the joystick and keyboard sessions. **a** Average decision accuracy (proportions correct) across participants. Error bars denote the standard errors of the means. **b** Average mean response times (RTs) across participants. Error bars denote the standard errors of the means. **c** Kolmogorov–Smirnov (K–S) statistics when comparing the RT distributions between response modalities. The scatter plot shows the K–S statistics in the difficult condition as a function of those in the easy condition. Each data point represents the correct (filled data point) or incorrect (open data point) trials of one participant. Linear regression lines are illustrated for correct (solid line) and incorrect (dashed line) trials

The behavioral performance of the four-alternative motion discrimination task was quantified by accuracy (proportions of correct responses; Fig. 2a) and mean RTs (Fig. 2b). We compared the behavioral performance between response modalities (joystick or keyboard), task difficulties (easy or difficult), and motion directions (up, down, left, or right) using three-way Bayesian and frequentist repeated measures ANOVAs. Across the two response modalities, participants showed decreased accuracy [BF_10_ = 5.112 × 10^30^; *F*(1, 20) = 292.709, *p* < .001] and increased mean RTs [BF_10_ = 1.458 × 10^18^; *F*(1, 20) = 63.163, *p* < .001] in the more difficult condition. We found compelling evidence against the main effect of response modality on accuracy [BF_10_ = 0.124; *F*(1, 20) = 0.083, *p* = *.*776] and weak evidence against the main effect of response modality on mean RT [BF_10_ = 0.560; *F*(1, 20) = 0.495, *p* = *.*490]. These results indicated similar behavioral performance between joystick and keyboard responses.

When comparing the behavioral performance between motion directions, compelling evidence against a main effect on accuracy emerged [BF_10_ = 0.185; *F*(2.248, 44.961) = 0.107, *p* = *.*357]. For mean RTs, the frequentist ANOVA suggested a significant main effect of motion direction [*F*(2.853, 57.052) = 3.021, *p* = *.*039], but this result was supported by neither post-hoc tests (*p > .*139 in all post-hoc comparisons, Bonferroni-corrected) nor a Bayesian ANOVA (BF_10_ = 0.305). Furthermore, there was a significant interaction between task difficulty and motion direction for accuracy [*F*(2.586, 51.718) = 6.317, *p* = *.*002], although this was again not supported by the Bayesian analysis (BF_10_ = 0.299). We found evidence against all the other interactions for both accuracy (BF_10_ < 0.179; *p > .*228) and mean RT (BF_10_ < 0.199; *p > .*083).

The results above suggested no systematic bias at the group level when comparing responses from a joystick and a keyboard. However, the consistency of behavioral performance between response modalities could vary between participants. For experiments with multiple response modalities, the researcher might want to confirm whether the consistency between response modalities is maintained across experimental conditions. This would allow, for example, a prescreening procedure to identify participants with high response consistency to be recruited for further experiments. Here we used K–S statistics to quantify the difference in individual participants’ RT distributions between the joystick and keyboard sessions in each difficulty condition, separately for correct and incorrect trials. There was strong evidence of a positive correlation between the K–S statistics of the easy and difficult conditions (correct trials, BF_10_ = 3.647 × 10^6^, *R* = .92, *p* < .001; incorrect trials, BF_10_ = 4,526.00, *R* = .82, *p* < .001; Fig. 2c). Therefore, the difference in behavioral performance between response modalities was consistent within participants across difficulty levels.

### Hierarchical DDM analyses

**Fig. 3:**
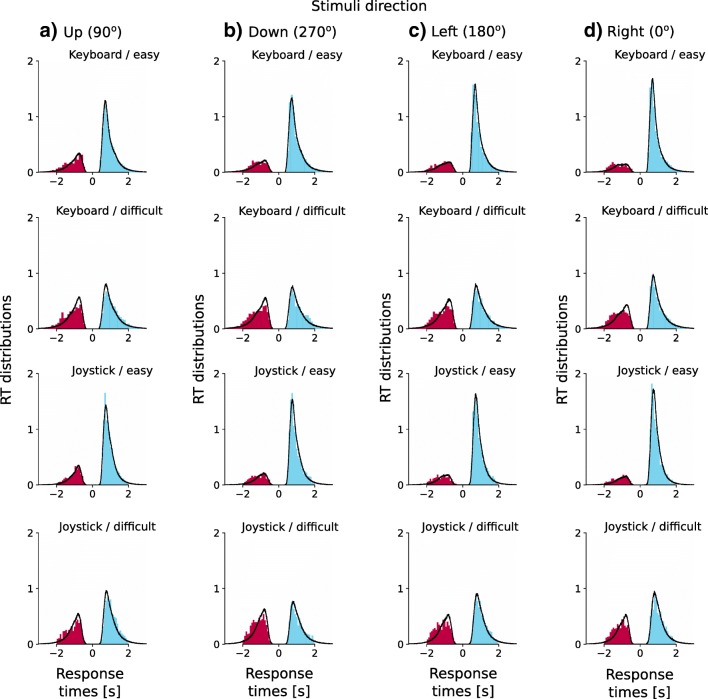
Posterior predictive response time (RT) distributions from the fitted drift-diffusion model. Each panel shows normalized histograms of the observed data (blue bars, correct responses; red bars, incorrect responses) and the model predictions (black lines) across participants. The RT distribution along the positive *x*-axis is from correct responses, and the areas under the curve on the positive *x*-axis correspond to the observed and predicted accuracy. The RT distribution along the negative *x*-axis is from error responses, and the areas under the curve on the negative *x*-axis correspond to the observed and predicted errors. The posterior predictions of the model were generated by averaging 500 simulations of the same amount of model predicted data that were observed in the experiment, using posterior parameter estimates

To compare the underlying decision-making processes between joystick and keyboard responses, we simplified the four-alternative motion discrimination task to a binary decision task (Churchland et al., 2008; see also the Drift-Diffusion Model section) and fitted the DDM to the behavioral data using the HDDM toolbox (Wiecki et al., 2013). The DDM decomposed individual participants’ behavioral data into model parameters for their latent psychological processes, and the HDDM toolbox allowed us to estimate the joint posterior estimates of model parameters using the hierarchical Bayesian approach. To evaluate the model fit, we generated model predictions by simulations with the posterior estimates of the model parameters. There was good agreement between the observed data and the model simulations across response modalities, task difficulties, and motion directions (Fig. 3).

With no a priori knowledge about the effect of response modality on the decision-making process, we allowed all model parameters to vary between joystick and keyboard responses: the boundary separation *a*, the mean drift rate *v*, the mean nondecision time *T*_er_, the trial-by-trial variability in drift rate *s*_*v*_, and the trial-by-trial variability in nondecision time *s*_*t*_(Table 1). The mean drift rate was further allowed to vary between task difficulties and motion directions. We performed Bayesian hypothesis testing on the posterior parameter estimates between response modalities (Bayarri & Berger, 2004; Gelman et al., 2014; Kruschke, 2015; Lindley, 1965). This analysis yielded 95% HDIs of the parameter differences between the joystick and keyboard sessions, as well as Bayesian *P* values *P*_*P|D*_(see the Data Analysis section for details).

**Table 1:** Posterior estimates of the hierarchical drift-diffusion model parameters (decision threshold *a*, mean drift rate *v*, nondecision time *T*_*er*_, trial-by-trial drift rate variability *s*_*v*_, and trial-by trial nondecision time variability *s*_*t*_)

			Joystick (mean ± *SD*)	Keyboard (mean ± *SD*)	95% HDI	*P*_*P|D*_
*a*			1.508 ± 0.072	1.572 ± 0.073	[– 0.270, 0.120]	.872
*v*	Easy	Up	1.694 ± 0.263	1.269 ± 0.260	[– 0.300, 1.144]	.720
		Down	1.765 ± 0.264	1.454 ± 0.261	[– 0.460, 0.999]	.810
		Left	2.169 ± 0.267	1.906 ± 0.260	[– 0.450, 1.020]	.789
		Right	2.351 ± 0.267	2.187 ± 0.262	[– 0.580, 0.880]	.863
	Difficult	Up	0.477 ± 0.257	0.291 ± 0.263	[– 0.526, 0.896]	.866
		Down	0.144 ± 0.262	0.202 ± 0.256	[– 0.822, 0.603]	.932
		Left	0.441 ± 0.261	0.216 ± 0.257	[– 0.529, 0.909]	.854
		Right	0.533 ± 0.263	0.597 ± 0.261	[– 0.769, 0.685]	.964
*T*_er_			0.613 ± 0.028	0.556 ± 0.028	[– 0.025, 0.130]	.658
*s*_*v*_			0.992 ± 0.047	0.916 ± 0.042	[– 0.039, 0.203]	.669
*s*_*t*_			0.268 ± 0.007	0.283 ± 0.007	[– 0.035, 0.004]	.641

The first two data columns show the posterior means and standard deviations of the parameters in the joystick and keyboard sessions. The 95% HDI column contains the 95% highest density intervals for the parameter differences between the joystick and keyboard sessions. *P*_*P|D*_ denotes the Bayesian *P* value for the parameters being equal between response modalities

For all the model parameters, we could not reject the null hypothesis that the posterior parameter estimates were practically equivalent between the joystick and keyboard sessions. The *P*_*P|D*_, which quantifies the probability that the model parameters are practically equivalent between the two conditions, ranged from .641 to .964 (Table 1). Therefore, we found no evidence to support that switching from keyboard to joystick altered the decision-making process. Next, because the mean drift rate is often assumed to increase with decreased task difficulty (Ratcliff & McKoon, 2008), we compared the drift rates averaged from the joystick and keyboard sessions between the easy and difficult conditions. As expected, the drift rate was larger in the easy than in the difficult condition in all motion directions (*up*: 95% HDI = [0.589, 1.613], *P*_*P|D*_ = 0; *down*: 95% HDI = [0.930, 1.958], *P*_*P|D*_ = 0; *left*: 95% HDI = [1.204, 2.227], *P*_*P|D*_ = 0; *right*: 95% HDI = [1.185, 2.214], *P*_*P|D*_ = 0).

### Additional measures from joystick trajectories

**Fig. 4:**
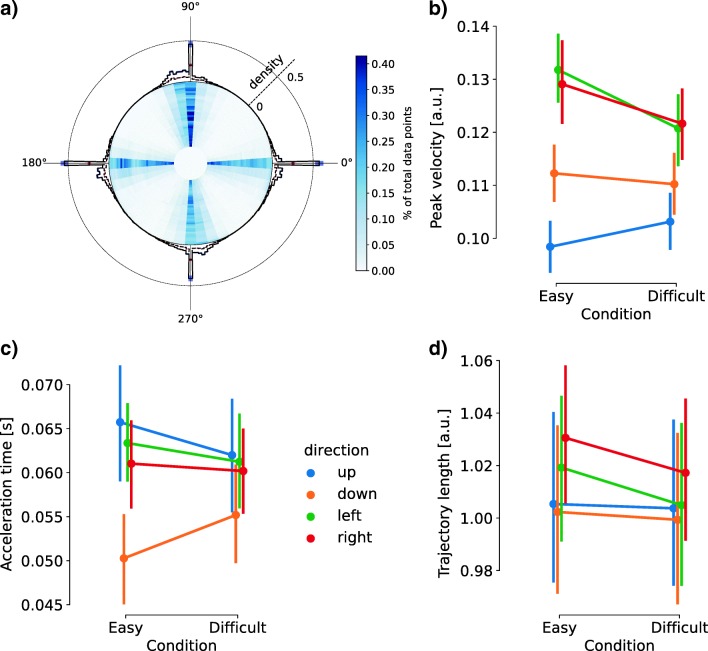
Measures from joystick trajectories. **a** Summary of movement trajectories and final positions. The heat map in the center represents the proportions of the total joystick positions across trials and participants. The histograms on the edge represent the distributions of final positions. Solid line represents correct responses. Dashed line represents incorrect responses. **b** Peak velocities of joystick movements, averaged across participants. **c** Mean acceleration times of joystick movements, averaged across participants. **d** Mean trajectory lengths, averaged across participants. The error bars denote the standard errors of the means. a.u., arbitrary units

In the joystick session, the participants’ movement trajectories were close to the four cardinal directions (Fig. 4a). Continuous movements with the joystick enabled us to acquire additional single-trial behavioral measures beyond those possible from simple key presses. We examined three such measures: peak velocity (Fig. 4b), acceleration time (Fig. 4c), and trajectory length (Fig. 4d). These additional joystick measures were analyzed subsequently to accuracy and RT. In the present study, we did not expect them to have a critical influence on the two primary behavioral measures. Hence, our analyses were focused on the effects of movement direction and task difficulty on the trajectory measures. However, we acknowledge that, in experiments with more complex movement trajectories, decisions might be more directly coupled to continuous motor responses (Song & Nakayama, 2009).

We calculated the action velocity as the rate of change of joystick position. There was a single peak of action velocity in each trial, consistent with the ballistic nature of the movement. We found strong evidence for the main effect of response direction on the peak velocity [Fig. 4b, BF_10_ = 3.900 × 10^24^; *F*(2.000, 40.002) = 39.25, *p* < .001], moderate evidence for the main effect of difficulty [BF_10_ = 4.612; *F*(1, 20) = 22.70, *p* < .001], and strong evidence for the interaction between direction and difficulty [BF_10_ = 58.433; *F*(2.841,56.813) = 30.58, *p* < .001].

We calculated the acceleration time as the latency between the RT and the time of peak velocity (Fig. 4c). There was strong evidence for the main effect of response direction [BF_10_ = 1,147.376; *F*(2.253, 45.05) = 4.741, *p* = *.*011]. We found moderate evidence against an effect of difficulty level [BF_10_ = 0.172; *F*(1, 20) = 0.178, *p* = *.*677]. Frequentist ANOVA showed a significant interaction between the response direction and difficulty level [*F*(2.853, 57.053) = 4.470, *p* = *.*008], which was not supported by the Bayes factor (BF_10_ = 0.256).

We calculated the trajectory length as the sum of the Euclidean distances between adjacent joystick positions in each trial (Fig. 4d). There was no compelling evidence for the main effect of response direction on trajectory length [BF_10_ = 1.759; *F*(3, 60) = 1.944, *p* = *.*151], nor a main effect of task difficulty [BF_10_ = 0.450; *F*(1, 20) = 3.171, *p* = *.*09]. The evidence against the interaction between direction and difficulty was strong [BF_10_ = 0.090; *F*(3, 60) = 0.978, *p* = *.*409].

In summary, the peak action velocity of joystick movements was affected by both action direction and task difficulty, and acceleration time was affected only by trajectory direction. There was no compelling evidence to support that trajectory length was affected by either action direction or task difficulty.

## Discussion

In the present study, we systematically compared the consistency between continuous and discrete responses during rapid decision-making. In a four-alternative motion discrimination task, joystick movements and key presses led to similar accuracy and mean RTs. Further modeling analysis with a hierarchical DDM showed no evidence in supporting a change of any model parameters between response modalities. Together, our findings provide evidence for the validity of using continuous joystick movement as a reliable response modality in behavioral experiments.

### Behavioral measures

In both joystick and keyboard sessions, participants had lower accuracy and longer mean RTs in the more difficult condition (i.e., lower motion coherence), in line with previous findings with similar tasks (Britten et al., 1992; Pilly & Seitz, 2009; Ramachandran & Anstis, 1983; Roitman & Shadlen, 2002). Using Bayesian statistics, we found evidence that response modality (joystick motion or key press) did not affect either accuracy or mean RT, confirming the validity of using joystick as a response device in decision-making tasks. Importantly, across participants, the difference in the RT distributions between response modalities was positively correlated between easy and difficult conditions. Therefore, participants with similar behavioral performance between response modalities maintained their consistency between experimental conditions.

Joystick positions estimated at a high sampling rate enabled additional behavioral measures beyond on/off key presses. In the present study, most of the movement trajectories were along the four cardinal directions (Fig. 4a). The averaged trajectory length was close to 1 (Fig. 4d), which was the shortest distance from the joystick’s neutral position to the maximum range, suggesting that the participants were able to make accurate and ballistic movements following the task instructions. Nevertheless, it is worth noting that the movement direction affected the peak velocity and acceleration time. This might have be due to the difference in upper limb muscle contractions when moving the joystick toward different directions (Oliver, Northey, Murphy, MacLean, & Sexsmith, 2011). Therefore, for future behavioral experiments relying on sensitive trajectory measures, we suggest extra caution be used in interpreting the effects of ergonomics and human motor physiology, especially for rapid movements, as in the present study. One potential solution would be to acquire baseline recordings of the movements to be expected during the experiment, which could then be used to compensate for measurement biases.

### Model-based measures

The DDM and other sequential-sampling models are commonly used to investigate the cognitive processes underlying rapid decision-making(Bogacz et al., 2006; Smith & Ratcliff, 2004). In the present study, the mean drift rate increased in the easier task condition, consistent with previous modeling results (Ratcliff & McKoon, 2008). The combination of posterior parameter estimation and Bayesian inference allowed us to obtain the probability of the parameter being practically equal, a more informative measure than frequentist *p* values (Kruschke, 2015). Although our results suggested that most parameter values had high probabilities to remain the same between response modalities (Table 1), we could not accept the null hypothesis for certain (which requires *P*_*P|D*_ = 1) and need more data to confirm the inference.

We highlighted two model parameters with low *P*_*P|D*_ values, which indicate that, with additional observed data from future experiments, the posterior model parameters might be in favor of the alternative hypothesis (i.e., a difference between response modalities). First, when switching from key presses to joystick movements, there was a small increase in the mean nondecision time (*P*_*P|D*_ = .658). Second, responding with a joystick resulted in a slightly decreased decision threshold (*P*_*P|D*_ = .872). Several previous studies have shown that instructing to respond faster or more accurately could efficiently modulate participants’ behavior (Beersma et al., 2003; Schouten & Bekker, 1967; Wickelgren, 1977). The decision threshold plays a substantial role under such speed–accuracy instructions (Mulder et al., 2013; Rae, Heathcote, Donkin, Averell, & Brown, 2014; Ratcliff & McKoon, 2008; Starns & Ratcliff, 2014; Zhang & Rowe, 2014): A decrease of threshold is accompanied with faster reaction speed and lower accuracy. If participants do implicitly trade accuracy for speed when switching from keyboard to joystick movements, this cognitive discrepancy needs to be considered when conducting experiments involving continuous responses. One hypothesis for this potential behavioral change is that continuous joystick movements allow participants to change or correct their responses later in a trial (Albantakis & Deco, 2009; Gallivan & Chapman, 2014; Gallivan, Logan, Wolpert, & Flanagan, 2016; Selen, Shadlen, & Wolpert, 2012), and this response flexibility may lead to reduced deliberation in initial movements.

The trial-by-trial variabilities in drift rate and nondecision time also had *P*_*P|D*_ values. Empirically, across-trial variability was introduced in DDM to improve the model fit to RT distributions (Ratcliff & McKoon, 2008), although the functional significance of these parameters to the decision process is still unclear. Across-trial variability in the drift rate produces different RT between correct and error trials (Ratcliff & Rouder, 1998), and across-trial variability in nondecision time accounts for the large variability in trials with short RTs across experimental conditions (Ratcliff & Tuerlinckx, 2002). These model parameters allow the DDM to account for the subtle differences in the shape of RT distributions between response modalities. Future studies could apply formal model comparison to evaluate the need of trial-by-trial variability in modeling joystick responses.

### The use of joystick and its validity

We aimed to establish the validity of joystick responses in rapid decision-making tasks. More specifically, we examined whether response modality (joystick movements vs. key presses) alters the raw behavioral measures (RT and accuracy) and underlying cognitive processes. We found that both behavioral measures and model parameters from cognitive modeling did not differ significantly between response modalities. In other words, using joystick movements to indicate choices of perceptual decisions elicit behavioral and cognitive characteristics similar to those from conventional key presses.

Motion discrimination based on random-dot kinematogram is a typical paradigm for simple decisions. The same computational mechanism of evidence accumulation has been suggested to account for the cognitive processes underlying a broad range of decision-making tasks, spanning across sensory modalities (O’Connell, Dockree, & Kelly, 2012) and cognitive domains (Gold & Shadlen, 2007). Therefore, we expect that the validity of joystick response established in the present study can be extended to experimental paradigms in which participants make rapid choices with motor actions (Ratcliff & McKoon, 2008).

The joystick as a response modality has been successfully applied in ageing and clinical populations, in which conventional key presses may be error-prone due to impaired dexterity. Both older and young adults can operate joysticks in visuomotor tasks with similar response patterns (Kramer, Larish, Weber, & Bardell, 1999). Previous studies showed that older adults can complete multiple hour-long cognitive training sessions with joystick responses, and the performance benefit persisted for six months after training (Anguera et al., 2013). In patients with neurodegenerative diseases, volitional joystick movements have been successfully used to examine the motor deficits and underlying neural abnormalities (Kew et al., 1993). This evidence suggested that the use of joystick can be well tolerated in older adults and patients.

In the present study, the participants did not report fatigue after joystick or keyboard sessions, which lasted approximately 45 min each. Other paradigms with longer experimental sessions and more intense joystick movements might impose a challenge to participants’ stamina. Nevertheless, it is possible to use measures from the continuous joystick recording (Kahol, Smith, Brandenberger, Ashby, & Ferrara, 2011) or concurrent physiological recording (Mascord & Heath, 1992) to identify the onset of fatigue prior to performance deterioration.

One might ask whether joystick responses provide any additional value over conventional key presses. Here, we showed that, even in simple ballistic movements, joystick-specific measures (e.g., action velocity) can be affected by the task difficulty, providing additional information on behavioral performance in addition to RT and accuracy. It is yet to be determined whether continuous responses provide information independent from discrete responses (Freeman, 2018; Freeman & Ambady, 2010; Stillman, Medvedev, & Ferguson, 2017). However, the capacity of recoding continuous responses via joysticks enables new experimental designs to probe the continuous interplay between action, perception and cognition. For example, the ongoing locomotion can modify the sensory information flow (Ayaz, Saleem, Schölvinck, & Carandini, 2013; Souman, Freeman, Eikmeier, & Ernst, 2010).

### Future directions

Three issues require further consideration. First, we only used a joystick to record movement trajectories, which is commonly used and widely available in behavioral testing labs. Many other devices are capable for recording continuous responses, such as computer mouse (e.g., Koop & Johnson, 2011), optic motion sensor (e.g., Chapman et al., 2010) and robotic arms (Abrams, Meyer, & Kornblum, 1990; Archambault, Caminiti, & Battaglia-Mayer, 2009; Burk, Ingram, Franklin, Shadlen, & Wolpert, 2014; Resulaj et al., 2009; van den Berg et al., 2016). The present study offered a comprehensive comparison between key presses and joystick movements, but the measures from other devices are yet to be validated. We also offered a practical solution to measure RT from joystick movement comparable to that from key presses, taking in to account the small resistive forces near the joystick’s neural position. To facilitate future research, we have made our data and analysis scripts openly available (https://osf.io/6fpq4).

Second, we instructed participants to make directional movements in the joystick session, which allows for intra-individual comparisons between response modalities. Motion trajectories suggested that the participants mainly made ballistic actions toward one of the four cardinal directions (Fig. 4a). One could explore the further potential of continuous responses in behavioral tasks, such as in response to a change of mind (Burk et al., 2014; Resulaj et al., 2009; van den Berg et al., 2016) or external distractions (Gallivan & Chapman, 2014).

Third, the DDM requires behavioral data to be presented as binary choices (Ratcliff & McKoon, 2008). To meet this constraint, we simplified our four-choice task data into correct and incorrect decisions, and incorrect responses contained errors toward three different directions from the correct motion direction. Our modeling results provided a good fit to the observed data. It would be useful to extend the analysis using other models that are designed for decision problems with multiple alternatives (Bogacz, Usher, Zhang, & McClelland, 2007; Brown & Heathcote, 2008; Usher & McClelland, 2001; Wong & Wang, 2006; Zhang & Bogacz, 2009), although a hierarchical Bayesian implementation of those more complex models is beyond the scope of the present study.

In conclusion, our results validated the joystick as a reliable device for continuous responses during rapid decision-making. As compared with key presses, the additional complexity and continuity associated with joystick movements did not affect raw behavioral measures such as accuracy and mean RT, as well as underlying decision-making processes. However, we highlighted the effects of movement direction on continuous trajectory measures. Researchers should be cautious when adopting experimental designs that require complex movement trajectories.

## Electronic supplementary material

Supplementary Fig. 1 The experimental setup and joystick positioning. Each participant was seated in front of the screen. Distance from the screen and head position were maintained using a chin rest. The seating height was adjusted to the most comfortable position, and the joystick was positioned to the right of the participant (A). The exact position of the device was adjusted to the most comfortable position. Participants were asked to hold the base of the joystick while responding. The keyboard was placed parallel to the screen to ensure that the arrow directions corresponded to the direction of the motion of the visual stimuli (B). (PDF 10871 kb)
