# Supplementary figures and images for: The validity and consistency of continuous joystick response in perceptual decision-making
Source: Behav Res Methods. 2019 Jul 3;52(2):681–93. doi: 10.3758/s13428-019-01269-3 (PMC7148284; doi:10.3758/s13428-019-01269-3)

Supplementary Figure 1

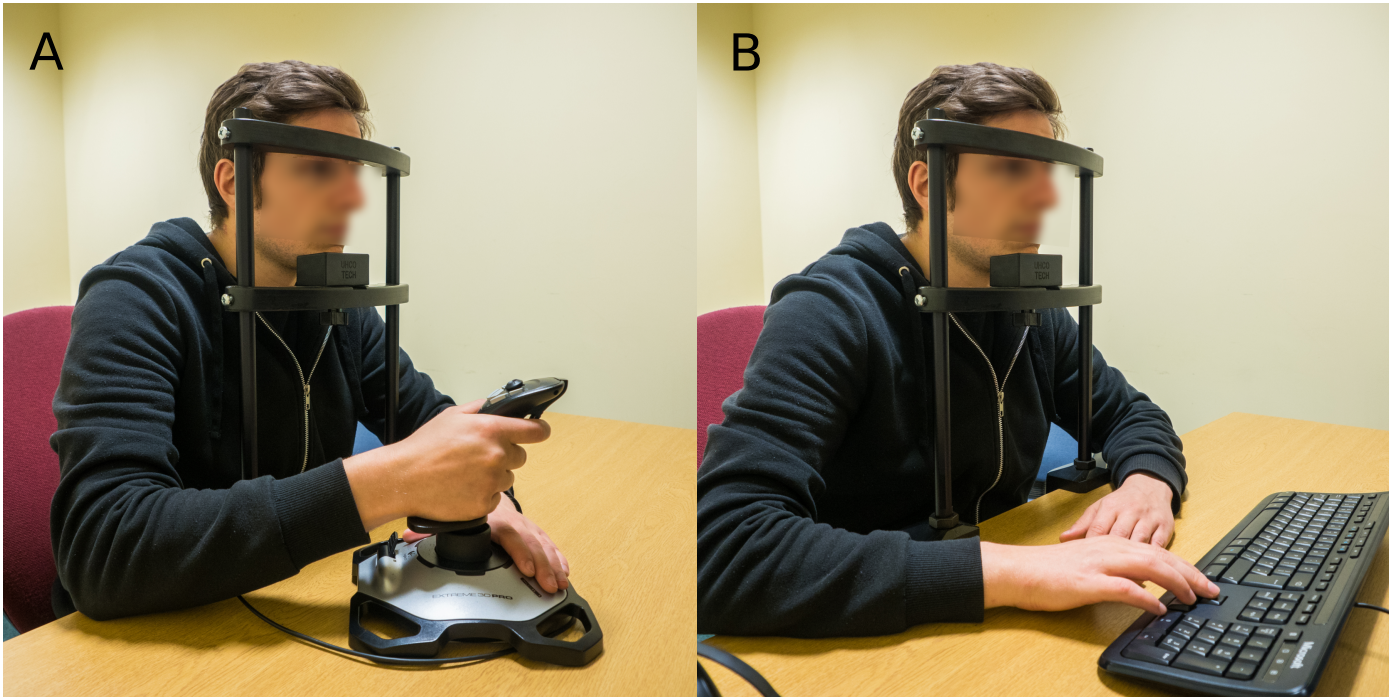

Supplement: Supplementary file 1 — The experimental setup and joystick positioning. Each participant was seated in front of the screen. Distance from the screen and head position were maintained using a chin rest. The seating height was adjusted to the most comfortable position, and the joystick was positioned to the right of the participant (A). The exact position of the device was adjusted to the most comfortable position. Participants were asked to hold the base of the joystick while responding. The keyboard was placed parallel to the screen to ensure that the arrow directions corresponded to the direction of the motion of the visual stimuli (B). (PDF 10871 kb) [file 13428_2019_1269_MOESM1_ESM.pdf]
